# Long-term risk of incident psoriasis in patients with irritable bowel syndrome: a large-scale prospective cohort study

**DOI:** 10.7189/jogh.16.04140

**Published:** 2026-08-07

**Authors:** Yuting Qiu, Yesheng Zhou, Si Liu, Qian Zhang, Shutian Zhang, Jing Wu, Shengtao Zhu, Shanshan Wu

**Affiliations:** 1 Department of Gastroenterology, Beijing Friendship Hospital, Capital Medical University, Beijing, China; 2 State Key Laboratory of Digestive Health, Beijing, China; 3 National Clinical Research Center for Digestive Disease, Beijing, China; 4 Beijing Key Laboratory of Early Gastrointestinal Cancer Medicine and Medical Devices, Beijing, China

**Keywords:** psoriasis, irritable bowel syndrome, cohort studies

## Abstract

**Background:**

While irritable bowel syndrome (IBS) and psoriasis share some pathophysiological features, it remains unclear whether they are associated with one another. Therefore, we aimed to investigate the long-term risk of incident psoriasis in a large prospective cohort of patients with IBS.

**Methods:**

We retrieved data on 437,170 participants from the UK Biobank without psoriasis at baseline. Using ICD-10 codes, we classified these participants into IBS and non-IBS groups based on recruitment status and assessed them for incident psoriasis at follow-ups as the primary outcome. We used multivariable Cox proportional hazards models to estimate adjusted hazard ratios (HRs) and 95% confidence intervals (CIs).

**Results:**

We identified 21,748 (5.0%) IBS patients among the 437,170 participants. There were 4325 (1.0%) incident psoriasis cases during a median follow-up period of 14.4 years, amounting to a cumulative incidence of 0.88% (95% CI = 0.73–1.02) in the IBS group *vs.* 0.69% (95% CI = 0.66–0.72) in the non-IBS group. Compared with non-IBS patients, patients with IBS had a 35.0% higher risk of developing psoriasis (HR = 1.35; 95% CI = 1.19–1.52). In subgroup analyses, a higher psoriasis risk associated with IBS was generally observed across age, sex, Townsend deprivation index, smoking status, non-steroidal anti-inflammatory drug use, C-reactive protein, and polygenic risk score of psoriasis subgroups.

**Conclusions:**

The IBS patients in our sample had an increased long-term risk of incident psoriasis. This finding highlights the importance of monitoring psoriasis in IBS patients and suggests a potential shared pathophysiological mechanism between the two conditions which may inform future preventive and therapeutic strategies.

Psoriasis is a chronic, immune-mediated inflammatory skin disease characterised by red, scaly plaques, commonly affecting functionally crucial areas such as elbows, knees, scalp, and lower back [1]. Its pathogenesis involves dysregulated T-cell activation, overproduction of proinflammatory cytokines, and genetic factors, underscoring its systemic inflammatory nature [2]. Beyond cutaneous manifestations, emerging evidence suggests that the condition is a multisystem disorder linked to increased risks of metabolic, cardiovascular, and gastrointestinal comorbidities [3]. Estimates indicate that psoriasis affects 1.92% adults in Western Europe [1].

Irritable bowel syndrome (IBS) is a common disorder of gut-brain interaction that manifests as recurrent abdominal pain and altered bowel habits without an identifiable organic cause [4]. It is becoming increasingly recognised for its extraintestinal manifestations, including systemic low-grade inflammation and a wide range of comorbidities, such as metabolic and autoimmune diseases, as well as depression and anxiety [5]. The condition is estimated to have a global prevalence of approximately 10% [4].

Recent evidence suggests that IBS and psoriasis may share overlapping pathophysiological pathways, including immune dysregulation, gut microbiota dysbiosis, and disturbances in the gut-skin axis [6]. Several case-control and cross-sectional studies have also reported positive associations between IBS and psoriasis [7,8]. However, these studies were limited by small sample sizes, insufficient adjustments for confounders, and methodological designs that hindered causal inference. To the best of our knowledge, no prospective cohort study to date has investigated the risk of incident psoriasis associated with IBS.

To address these gaps, we aimed to examine the association between IBS and the risk of incident psoriasis in a large, population-based, prospective cohort study.

## METHODS

This study is a secondary analysis of existing UK Biobank data and was reported in accordance with the GRABDROP guidelines. Detailed responses to the GRABDROP items are provided in Table S1 in the **Online Supplementary Document**.

### Study population

**Figure 1:**
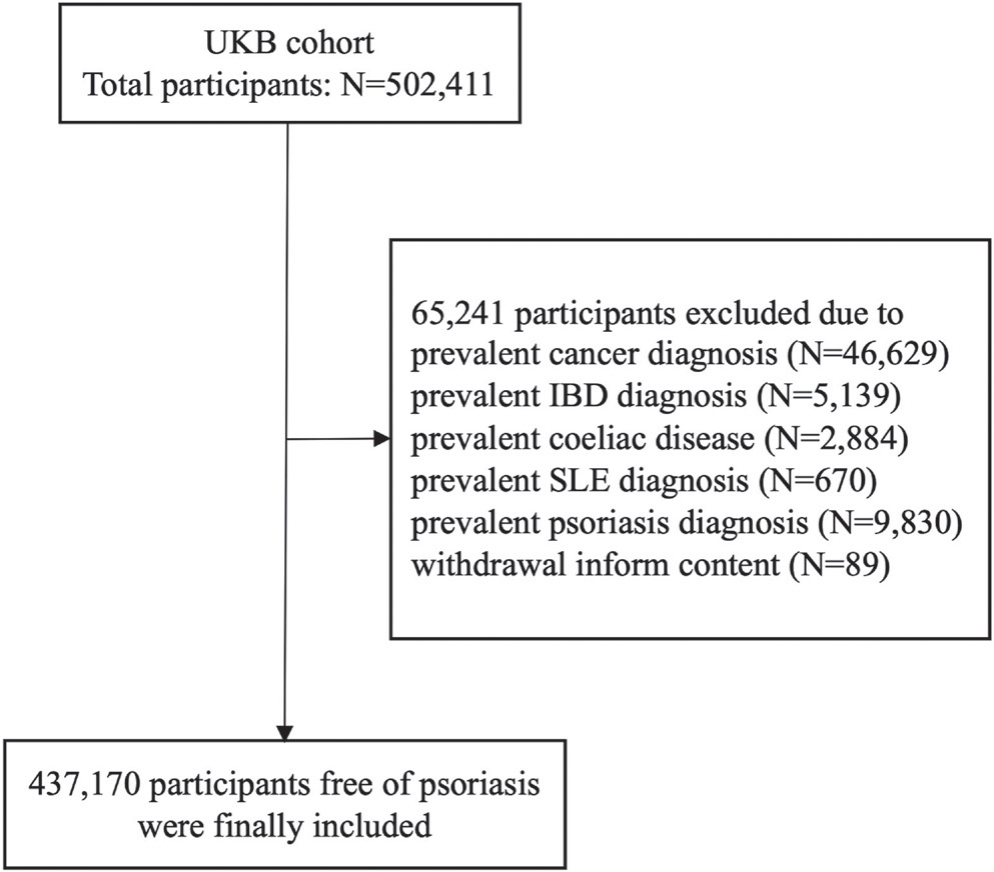
Flowchart of the study population. IBD – inflammatory bowel disease, SLE – systemic lupus erythematosus, UKB – UK Biobank.

We retrieved data from the UK Biobank, a large population-based cohort that enrolled individuals aged 37–73 years across the UK between 2006 and 2010. At recruitment, all participants provided written informed consent, completed baseline questionnaires, and underwent a range of physical and biochemical tests. All participants enrolled in the UK Biobank were initially included (n = 502,411). We excluded participants with a diagnosis of cancer, inflammatory bowel disease (IBD), coeliac disease, systemic lupus erythematosus (SLE), or psoriasis at baseline, as well as those who had withdrawn their informed consent. After these exclusions, participants were included in the final analytical cohort. We ascertained all disease diagnoses using codes from the ICD-10 (**Figure 1**). Follow-up commenced on the date of enrolment and continued until the first diagnosis of psoriasis, death, loss to follow-up, or the end of the study on 30 September 2023, whichever occurred first.

### Ascertainment of prevalent IBS

We determined the participants’ baseline IBS status (ICD-10 code K58) through self-reported or linked primary care and/or hospital admission records across the UK. We categorised participants with any diagnosis of IBS before or at enrolment into the IBS group, and those without an IBS diagnosis, regardless of whether IBS occurred during follow-up, as the non-IBS (*i.e.* reference) group.

### Ascertainment of incident psoriasis

The primary outcome was incident psoriasis (ICD-10 code L40) during follow-up, with an endpoint follow-up date of 30 September 2023. We identified incident psoriasis diagnoses through self-report or linkage to primary care and/or hospital admission data.

### Covariates

We selected baseline covariates based on previous epidemiological evidence and data availability [2,7,9,10]: age (in years, treated as continuous variable); sex (male, female), ethnicity (white, non-white); education level (university, non-university), Townsend deprivation index (TDI), calculated using the preceding national census output areas as a socioeconomic deprivation measurement and divided into quartiles; body mass index (BMI), categorised as <18.5, 18.5–24.9, 25.0–29.9, ≥30 kg/m2; alcohol use (never, previous, current); smoking status (never, previous, current); physical activity based on the International Physical Activity Questionnaire (low, moderate, high) [11]; type 2 diabetes mellitus (yes, no); self-reported non-steroidal anti-inflammatory drug (NSAID) use, *e.g.* aspirin, ibuprofen, nurofen, or paracetamol (yes, no); and C-reactive protein (CRP) values (continuous). All of the data above was obtained from the records in the UK Biobank. The median value of TDI (−2.12) was set as the cutoff, with TDI ≤ −2.12 considered the low group and TDI > −2.12 the high group. The median value of psoriasis PRS (−0.35) was set as the cutoff value, with PRS ≤ −0.35 considered the low-risk group and PRS > −0.35 the high-risk group. 

We additionally adjusted for the presence of depression (yes, no) and anxiety (yes, no) in the sensitivity analysis. We also accounted for genetic susceptibility to psoriasis by adjusting for a standard polygenic risk score (PRS) from the official UK Biobank PRS Release [12]. To assess the influence of dietary patterns, we defined them based on the following seven dietary components (fruits ≥3 servings/d; vegetables ≥3 servings/d; fish ≥2 servings/week; processed meats ≤1 serving/week; unprocessed red meats ≤1.5 servings/week; whole grains ≥3 servings/d; refined grains ≤1.5 servings/d) and classified them into two levels (unhealthy and healthy), with a diet that received a score of ≥4 being considered healthy. Furthermore, we calculated IBS duration as the time interval between the first recorded IBS diagnosis and the baseline assessment date.

### Statistical analysis

We employed Cox proportional hazards models to investigate the risk of incident psoriasis associated with IBS. In addition to the univariable analysis, we applied three multivariable models, where we adjusted model 1 for age and sex; model 2 for the two variables in model 1 and ethnicity, education level, TDI, BMI, alcohol drinking, smoking status, physical activity; and model 3 for all variables in model 2 plus type 2 diabetes mellitus, NSAID use, and CRP. We evaluated the proportional hazards assumption using Schoenfeld residual tests. Given the very small percentage of missing data for most covariates, missing values were handled using the missing–indicator method, whereby missing values were coded as a separate category for the corresponding covariate.

We conducted subgroup analyses by age (<60, ≥60 years), sex (male, female), TDI (low, high), BMI (<25, ≥25 kg/m2), drinking status (never/previous, current), smoking status (never, previous/current), physical activity (low/moderate, high), depression/anxiety (no, yes), NSAID use (no, yes), CRP (≤8 mg/L, >8 mg/L), and PRS of psoriasis (low, high). We applied Benjamini–Hochberg false discovery rate (FDR) correction to account for multiple testing and examined potential effect modifications by including a cross-product interaction term as an additional independent variable in model 3. To assess whether IBS duration was associated with the risk of incident psoriasis, we categorised participants into those with ≤10 years and >10 years of IBS duration and performed Cox proportional hazards analysis.

We performed several sensitivity analyses to test the robustness of our findings. First, we excluded participants diagnosed with psoriasis within one or two years after recruitment to minimise reverse causation and those with NSAID use to eliminate the influence of NSAID use on psoriasis incidence. Furthermore, we applied the Fine–Gray competing risk model, with death and loss to follow-up considered as competing events; with additional adjustments for anxiety/depression, PRS of psoriasis, healthy diet score, or dietary patterns; after excluding self-reported psoriasis cases; and after addressing missing covariate data using multiple imputation, implemented with the ‘mice’ package in *R*.

We calculated the events per variable (EPV) ratio to evaluate model stability and the risk of overfitting, with an EPV>10 considered sufficient for robust estimation in multivariable Cox models. We conducted statistical analyses using SAS, version 9.4 (SAS Institute, Cary, NC, USA) and *R*, version 4.3.0 (R Core Team, Vienna, Austria). A two-tailed *P*-value <0.05 was considered statistically significant.

## RESULTS

### Baseline characteristics

All participants enrolled in the UK Biobank were initially included (n = 502,411). We included participants who were free of psoriasis at baseline and excluded those diagnosed with cancer (n = 46,629), IBD (n = 5139), coeliac disease (n = 2884), or SLE (n = 670), as well as those who withdrew from the study at or before enrolment (n = 89). Finally, we included 437,170 participants in the analysis. Among these 437,170 participants (53.3% female; mean age of 56.20 years, standard deviation = 8.11), there were 21,748 (5.0%) cases of IBS (**Table 1**). Compared to non-IBS patients, those with IBS were younger, predominantly female, had higher proportions of NSAID use, higher CRP levels, and a higher prevalence of anxiety and depression.

**Table 1:** Baseline characteristics according to prevalent IBS status*

		Prevalent IBS status		
**Characteristics**	**Overall (n = 437,170)**	**No (n = 415,422)**	**Yes (n = 21,748)**	***P*-value**
Age in years, x̄ (SD)	56.20 (8.11)	56.21 (8.12)	55.92 (7.92)	<0.001
Sex				<0.001
*Male*	204,162 (46.70)	198,334 (47.74)	5828 (26.80)	
*Female*	233,008 (53.30)	217,088 (52.26)	15,920 (73.20)	
Ethnicity				<0.001
*Non-white*	25,980 (5.94)	25,170 (6.06)	810 (3.72)	
*White*	409,517 (93.67)	388,653 (93.56)	20,864 (95.94)	
*Unknown*	1673 (0.38)	1599 (0.38)	74 (0.34)	
TDI, x̄ (SD)	−1.28 (3.10)	−1.28 (3.10)	−1.28 (3.11)	0.869
*Q1 (≤−3.63)*	109,285 (25.00)	103,710 (24.96)	5575 (25.63)	0.022
*Q2 (−3.63 to −2.12)*	109,249 (25.00)	103,741 (24.97)	5508 (25.33)	
*Q3 (−2.12 to 0.57)*	109,107 (24.96)	103,747 (24.97)	5360 (24.65)	
*Q4 (>0.57)*	108,975 (24.93)	103,690 (24.96)	5285 (24.30)	
*Unknown*	554 (0.13)	534 (0.13)	20 (0.09)	
BMI (kg/m2), x̄ (SD)	27.44 (4.79)	27.44 (4.78)	27.31 (5.09)	<0.001
*Normal (18.5–24.9 kg/m2)*	136,339 (31.19)	128,955 (31.04)	7384 (33.95)	<0.001
*Underweight (<18.5 kg/m2)*	2194 (0.50)	2005 (0.48)	189 (0.87)	
*Overweight (25.0–29.9 kg/m2)*	187,059 (42.79)	178,407 (42.95)	8652 (39.78)	
*Obesity (≥30 kg/m2)*	108,851 (24.90)	103,433 (24.90)	5418 (24.91)	
*Unknown*	2727 (0.62)	2622 (0.63)	105 (0.48)	
Education				<0.001
*Non-university*	286,133 (65.45)	271,346 (65.32)	14,787 (67.99)	
*University*	142,100 (32.50)	135,480 (32.61)	6620 (30.44)	
*Unknown*	8937 (2.04)	8596 (2.07)	341 (1.57)	
Smoking				<0.001
*Never*	241,091 (55.15)	228,828 (55.08)	12,263 (56.39)	
*Previous*	147,518 (33.74)	140,290 (33.77)	7228 (33.24)	
*Current*	45,973 (10.52)	43,831 (10.55)	2142 (9.85)	
*Unknown*	2588 (0.59)	2473 (0.60)	115 (0.53)	
Alcohol drinking				<0.001
*Never*	19,539 (4.47)	18,500 (4.45)	1039 (4.78)	
*Previous*	15,305 (3.50)	14,229 (3.43)	1076 (4.91)	
*Current*	400,834 (91.69)	381,265 (91.78)	19,569 (89.98)	
*Unknown*	1492 (0.34)	1428 (0.34)	64 (0.29)	
IPAQ				<0.001
*Low*	61,824 (14.14)	58,513 (14.09)	3311 (15.22)	
*Moderate*	136,443 (31.21)	129,865 (31.26)	6578 (30.25)	
*High*	137,944 (31.55)	131,727 (31.71)	6217 (28.59)	
*Unknown*	100,959 (23.09)	95,317 (22.94)	5642 (25.94)	
T2DM	11,271 (2.58)	10,743 (2.59)	528 (2.43)	0.158
NSAID use	171,657 (39.27)	161,368 (38.84)	10,289 (47.31)	<0.001
CRP (mg/L), Mdn (IQR)	1.30 (0.65–2.70)	1.30 (0.64–2.69)	1.37 (0.66–2.88)	<0.001
Anxiety	16,418 (3.76)	14,067 (3.39)	2351 (10.81)	<0.001
Depression	35,215 (8.06)	31,315 (7.54)	3900 (17.92)	<0.001

BMI – body mass index, CRP – C-reactive protein, IBS – irritable bowel syndrome, IQR – interquartile range, IPAQ – international physical activity questionnaire, Mdn – median, NSAID – non-steroidal anti-inflammatory drug, Q – quartile, SD – standard deviation, T2DM – type 2 diabetes mellitus, TDI – Townsend deprivation index, x̄ – mean

^*^Categorical variables are presented as frequencies (%) unless specified otherwise.

### Baseline IBS and risk of incident psoriasis

We identified 4325 incident psoriasis cases during a follow up of 14.4 years or 6,125,039 person-years. The cumulative incidence of psoriasis over this period was 0.88% (95% CI = 0.73–1.02) in the IBS group *vs.* 0.69% (95% CI = 0.66–0.72) in the non-IBS group. Individuals with IBS had a relative 35.0% higher risk of developing psoriasis than those without IBS (hazard ratio (HR) = 1.35; 95% CI = 1.19–1.52) (**Table 2**). The proportional hazards assumption was satisfied for the model (*P*-value for Schoenfeld’s test = 0.058), while the EPV ratio of 154 indicated excellent model stability.

**Table 2:** Risk of incident psoriasis associated with baseline IBS*

IBS status	No	Yes	*P*-value
Number of participants	415,422	21,748	
Number of incidents of psoriasis	4,033	292	
Follow-up, person-years	5,817,643	307,396	
Follow-up in years, Mdn (IQR)	14.4 (13.6–15.1)	14.4 (13.7–15.2)	
Incident psoriasis, HR (95% CI)			
*Unadjusted Model*	ref	1.35 (1.20–1.52)	<0.001
*Adjusted Model 1*	ref	1.41 (1.25–1.59)	<0.001
*Adjusted Model 2*	ref	1.38 (1.22–1.55)	<0.001
*Adjusted Model 3*	ref	1.35 (1.19–1.52)	<0.001

BMI – body mass index, CRP – C-reactive protein, HR – hazard radio, IBS – irritable bowel syndrome, IQR – interquartile range, IPAQ – International Physical Activity Questionnaire, Mdn – median, NSAID – non-steroidal anti-inflammatory drug, Q – quartile, ref – reference, SD – standard deviation, T2DM – type 2 diabetes mellitus, TDI – Townsend deprivation index

*Model 1 was adjusted for age and sex; model 2 was further adjusted for ethnicity, education level, TDI, BMI, alcohol drinking, smoking status, and IPAQ score; model 3 was further adjusted from model 2 for T2DM, NSAID use, and CRP.

### Subgroup analyses

**Figure 2:**
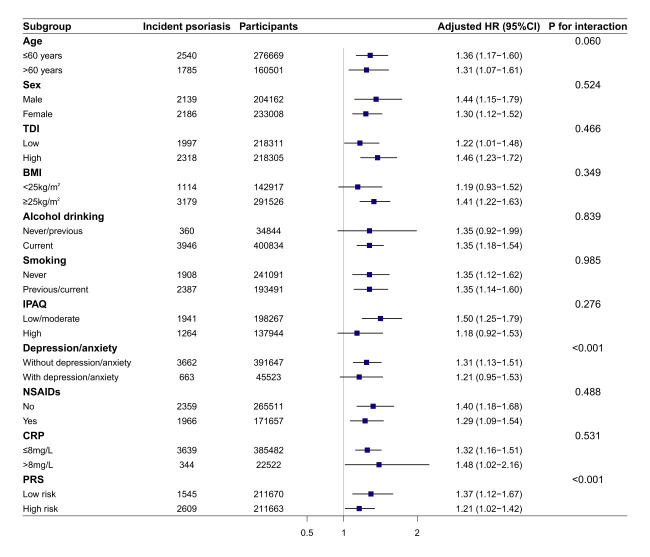
Subgroup analysis for the association between baseline IBS and incident psoriasis. The median value of TDI (−2.12) was set as the cutoff, with TDI≤−2.12 considered the low group and TDI>−2.12 the high group. The median value of psoriasis PRS (−0.35) was set as the cutoff value, with PRS≤−0.35 considered the low-risk group and PRS>−0.35 the high-risk group. All adjusted HRs were calculated by adjusting for the following covariates: age, sex, ethnicity, educational level, TDI, BMI, alcohol drinking, smoking status, IPAQ, T2DM, NSAID use and CRP. BMI – body mass index, CI – confidence interval, CRP – C-reactive protein, HR – hazard ratio, IBS – irritable bowel syndrome, IPAQ – International Physical Activity Questionnaire, NSAID – non-steroidal anti-inflammatory drug, PRS – polygenic risk score, TDI – Townsend deprivation index, T2DM – type 2 diabetes mellitus.

We generally observed higher psoriasis risk associated with IBS across age, sex, TDI, smoking status, NSAID use, CRP, and psoriasis PRS subgroups, with a significantly higher psoriasis risk in those without depression/anxiety (*P*-value for interaction <0.001) and those with low psoriasis PRS (*P*-value for interaction <0.001) (**Figure 2**; Tables S2–12 in the **Online Supplementary Document**). The results remained consistent after FDR correction. All subgroup analyses maintained an EPV > 10 (range = 12.3–140.9), indicating the HRs were precise.

In the duration-stratified analysis, both IBS duration ≤10 years and >10 years were associated with an increased risk of incident psoriasis compared with participants without IBS (Table S13 in the **Online Supplementary Document**). In the fully adjusted model, the HR was 1.45 (95% CI = 1.22–1.71) for participants with IBS duration ≤10 years and 1.26 (95% CI = 1.06–1.51) for those with IBS duration >10 years *(P <* .001).

### Sensitivity analyses

Sensitivity analyses consistently supported the primary findings across different definitions of incident psoriasis, after the exclusion of NSAID users, after performing competing-risk models, and after adjustments for various covariates, including anxiety/depression, PRS of psoriasis, healthy diet score or diet pattern, exclusion of self-reported psoriasis cases, and multiple imputation for missing covariate data (**Table 3**).

**Table 3:** Sensitivity analysis regarding the risk of incident psoriasis associated with baseline IBS*

IBS status	Number of incidents of psoriasis	Number of participants	HR (95% CI)	*P*-value
Sensitivity analysis 1: excluding psoriasis participants diagnosed within 1 year after baseline (n = 437 168)				
*No*	4031	415 420	ref	
*Yes*	292	21 748	1.35 (1.19–1.53)	<0.001
Sensitivity analysis 2: excluding psoriasis participants diagnosed within two years after baseline (n = 437 164)				
*No*	4027	415 416	ref	
*Yes*	292	21 748	1.35 (1.19–1.53)	<0.001
Sensitivity analysis 3: excluding participants with NSAID use (n = 265 511)				
*No*	2219	254 052	ref	
*Yes*	140	11 459	1.40 (1.18–1.68)	<0.001
Sensitivity analysis 4: competing risk model (n = 437 170; number of competing events = 35 202)				
*No*	4033	415 422	ref	
*Yes*	292	21 748	1.38 (1.22–1.55)	<0.001
Sensitivity analysis 5: additionally adjusted for depression and anxiety (n = 437 170)				
*No*	4033	415 422	ref	
*Yes*	292	21 748	1.26 (1.11–1.43)	<0.001
Sensitivity analysis 6: additionally adjusted for psoriasis PRS (n = 437 170)				
*No*	4033	415 422	ref	
*Yes*	292	21 748	1.35 (1.19–1.53)	<0.001
Sensitivity analysis 7: additionally adjusted for healthy diet score (n = 437 170)				
*No*	4033	415 422	ref	
*Yes*	292	21 748	1.35 (1.19–1.52)	<0.001
Sensitivity analysis 8: additionally adjusted for healthy diet pattern (n = 437 170)				
*No*	4033	415 422	ref	
*Yes*	292	21 748	1.35 (1.19–1.52)	<0.001
Sensitivity analysis 9: additionally excluding self-reported cases (n = 436 969)				
*No*	3846	415 235	ref	
*Yes*	278	21 734	1.37 (1.21–1.55)	<0.001
Sensitivity analysis 10: Multiple imputation for missing covariates (n = 437 170)				
*No*	4033	415 422	ref	
*Yes*	292	21 748	1.36 (1.20–1.53)	<0.001

BMI – body mass index, CI – confidence interval, CRP – C-reactive protein, IBS – irritable bowel syndrome, HR – hazard ratio, IQR – interquartile range, IPAQ – international physical activity questionnaire, MD – median, NSAID – non-steroidal anti-inflammatory drug, PRS – polygenic risk score, Q – quartile, ref – reference, SD – standard deviation, T2DM – type 2 diabetes mellitus, TDI – Townsend deprivation index, x̄ – mean

*All adjusted HRs were calculated by adjusting the following covariates: age, sex, ethnicity, education level, TDI, BMI, alcohol drinking, smoking status, IPAQ, T2DM, NSAID use, and CRP.

## DISCUSSION

We found that individuals with IBS had a 35.0% higher risk of developing psoriasis compared to those without IBS. To date, no prospective cohort study has examined the association between IBS and the risk of incident psoriasis. A cross-sectional study from Australia and a case-control study from Israel both reported significant associations between IBS and psoriasis, with odds ratios of 1.40 to 2.22, which align with our findings [7,8]. Additionally, psoriasis is frequently comorbid with other gastrointestinal immune-inflammatory conditions, such as inflammatory bowel disease, celiac disease, and non-alcoholic fatty liver disease, also supporting our findings [13,14].

### Potential mechanisms

Several biological mechanisms may account for the association between IBS and the development of psoriasis. First, chronic low-grade systemic inflammation in IBS is characterised by elevated concentrations of pro-inflammatory cytokines, such as tumour necrosis factor-α and interleukin-23, which may contribute to the initiation or aggravation of psoriatic lesions [15]. Second, IBS-related immune dysregulation, including abnormal T-cell activation and amplified Th1 and Th17 responses, mirrors the immunological profile observed in psoriasis, indicating shared inflammatory pathways [16,17]. Third, alterations in the gut microbial composition commonly observed in IBS patients may compromise intestinal barrier integrity, facilitating the translocation of microbial products into the systemic circulation and triggering immune activation that contributes to psoriatic pathogenesis [18]. Fourth, dysregulation of the gut-skin axis, driven by aberrant gut physiology in IBS, may disrupt immune and epithelial homeostasis, thereby increasing susceptibility to cutaneous inflammation [19]. Finally, psychological stress and emotional distress, frequently comorbid with IBS, may activate the hypothalamic-pituitary-adrenal axis and associated neuroimmune pathways, further amplifying systemic inflammation and potentially exacerbating or precipitating psoriasis [20,21].

Significant interactions revealed a more pronounced association between IBS and psoriasis risk among individuals with low genetic risk or without depression/anxiety. This suggests that the dominant influence of genetic and psychiatric factors might mask the relative contribution of IBS [2,22], which would become more discernible in the absence of these potent risk factors. Additionally, we observed a higher risk of incident psoriasis among participants with a shorter duration of IBS (≤10 years) compared with those with longer disease duration. One possible explanation is that the early phase of IBS may be characterised by more active immune dysregulation and gut barrier dysfunction, which could potentially contribute to the development of immune-mediated diseases such as psoriasis. As IBS becomes longstanding, it may reach a relatively stable state, potentially attenuating the associated risk of incident psoriasis.

### Implications for clinical practice and future research

We found a statistically significant increase in the relative risk of psoriasis among patients with IBS. However, it should be emphasised that the absolute risk difference remains small, consistent with the low incidence of psoriasis, and the public health impact of this association should be interpreted with caution. Nevertheless, considering that psoriasis is an incurable, chronic inflammatory disease with a profound impact on quality of life, identifying subpopulations at a marginally higher absolute risk may still facilitate personalised screening and early dermatological consultation, thereby improving prognosis.

Future studies should adopt a longitudinal design to elucidate the temporal sequence and potential causal relationships between IBS and psoriasis. Simultaneously, there is a need for further mechanistic investigations into the roles of immune dysregulation, the gut-skin axis, and gut microbiome alterations in the pathophysiology of both disorders. Interventional trials assessing whether strategies targeting gut health, systemic inflammation, or psychological stress can mitigate the risk of psoriasis in patients with IBS would provide valuable insights for preventive and therapeutic strategies.

### Strengths and limitations

Our study had several strengths, including its large cohort and a 14.4-year follow-up period. To the best of our knowledge, it is the first to explore the association between IBS and the risk of incident psoriasis. Furthermore, we conducted a series of subgroup and sensitivity analyses to ensure the robustness and reliability of our findings.

However, several limitations warrant consideration. First, although the use of ICD-10 codes for IBS and psoriasis diagnosis was epidemiologically justified, asymptomatic or mildly symptomatic individuals who did not seek medical care may have been missed. Second, patients with IBS may exhibit increased healthcare utilisation, potentially leading to a higher likelihood of psoriasis detection. To address this, we conducted sensitivity analyses excluding psoriasis cases diagnosed within one to two years after baseline, and our results remained consistent. Moreover, as it is characterised by persistent and visible cutaneous lesions, psoriasis typically necessitates dermatological intervention regardless of baseline healthcare-seeking propensity. Thus, the impact of detection bias on the overall validity of our findings is limited. Third, due to the lack of baseline IBS subtype data, we were unable to examine the association between IBS subtypes and incident psoriasis. Given that distinct IBS subtypes may possess varying systemic inflammatory profiles, this lack of specificity may limit the biological interpretation of our findings, leaving a need for future research with detailed clinical phenotyping. Fourth, despite adjusting for multiple confounders, the potential influence of residual confounding, such as use of antibiotics, corticosteroids, or probiotics, cannot be entirely ruled out due to the unavailability of this data in the cohort. Additionally, data on IBS severity were not available, which precluded analyses stratified by disease severity. Finally, the predominantly white ethnic composition of our cohort may limit the generalisability of our findings to other racial and ethnic populations.

## CONCLUSIONS

Our study underscores the importance of monitoring for psoriasis in patients with IBS and suggests shared pathophysiological mechanisms between the two conditions. While these findings may inform the development of future preventive and therapeutic strategies, further research in ethnically and geographically diverse populations is still warranted to validate these results and elucidate the underlying biological pathways.

## Additional material


Online Supplementary Document

## Data Availability

**Data availability:** All data relevant to the study were used in the UK Biobank Resource under application number 74444. No additional data available.
