# Supplementary material for: Long-term risk of incident psoriasis in patients with irritable bowel syndrome: a large-scale prospective cohort study
Source: J Glob Health. 2026 Aug 7;16:04140. doi: 10.7189/jogh.16.04140 (PMC13449552; doi:10.7189/jogh.16.04140)
Supplement: Supplementary file 1 [file jogh-16-04140-s001.zip › jogh-16-04140-s001.pdf]

Supplement to: Qiu Y, Zhou Y, Liu S, Zhang Q, Zhang S, Wu J, Zhu S, Wu S. Long-term risk of incident psoriasis in patients with irritable bowel syndrome: a large-scale prospective cohort study. J Glob Health. 2026;16:04140.

Table S2. Association between baseline IBS and risk of incident psoriasis by age....................... 2
Table S3. Association between baseline IBS and risk of incident psoriasis by sex....................... 3
Table S4. Association between baseline IBS and risk of incident psoriasis by TDI...................... 4
Table S5. Association between baseline IBS and risk of incident psoriasis by BMI..................... 5
Table S6. Association between baseline IBS and risk of incident psoriasis by alcohol drinking..... 6
Table S7. Association between baseline IBS and risk of incident psoriasis by smoking status....... 7
Table S8. Association between baseline IBS and risk of incident psoriasis by IPAQ.................... 8
Table S9. Association between baseline IBS and risk of incident psoriasis by depression/anxiety.. 9
Table S10. Association between baseline IBS and risk of incident psoriasis by NSAID use........ 10
Table S11. Association between baseline IBS and risk of incident psoriasis by CRP................. 11
Table S12. Association between baseline IBS and risk of incident psoriasis by PRS.................. 12
Table S13. Risk of incident psoriasis associated with IBS duration... 13

Table S2. Association between baseline IBS and risk of incident psoriasis by age.

Subgroup	IBS		*P*-value	P_{FDR}
	No	Yes		
**Age ≤ 60 years**				<0.001
No. of participants	262,393	14,276		
No. of incident psoriasis	2,355	185		
**Hazard ratio for incident psoriasis (95% CI, *P*-value)**				
Unadjusted Model	reference	1.43 (1.23-1.66)	<0.001	
Adjusted Model 1	reference	1.45 (1.25-1.69)	<0.001	
Adjusted Model 2	reference	1.41 (1.21-1.64)	<0.001	
Adjusted Model 3	reference	1.36 (1.17-1.60)	<0.001	
**Age > 60 years**				0.014
No. of participants	153,029	7,472		
No. of incident psoriasis	1,678	107		
**Hazard ratio for incident psoriasis (95% CI, *P*-value)**				
Unadjusted Model	reference	1.27 (1.04-1.54)	0.018	
Adjusted Model 1	reference	1.35 (1.11-1.64)	0.003	
Adjusted Model 2	reference	1.32 (1.09-1.61)	0.005	
Adjusted Model 3	reference	1.31 (1.07-1.61)	0.010	

Note: Adjusted Model 1: Age and sex were adjusted; Adjusted Model 2: Ethnicity, educational level, TDI, BMI, alcohol drinking, smoking status and IPAQ were additionally adjusted; Adjusted Model 3: T2DM, NSAID use and CRP were additionally adjusted.

Abbreviations: IBS, irritable bowel syndrome; FDR, false discovery rate; CI, confidence interval; TDI, Townsend Deprivation Index; BMI, Body Mass Index; IPAQ, International Physical Activity Questionnaire; T2DM, type 2 diabetes mellitus; NSAID, non-steroidal anti-inflammatory drug; CRP, C-reactive protein.

Table S3. Association between baseline IBS and risk of incident psoriasis by sex.

Subgroup	IBS		*P*-value	P_{FDR}
	No	Yes		
**Male**				0.003
No. of participants	198,334	5,828		
No. of incident psoriasis	2,052	87		
**Hazard ratio for incident psoriasis (95% CI, *P*-value)**				
Unadjusted Model	reference	1.41 (1.14-1.75)	0.002	
Adjusted Model 1	reference	1.42 (1.15-1.76)	0.001	
Adjusted Model 2	reference	1.42 (1.15-1.76)	0.001	
Adjusted Model 3	reference	1.44 (1.15-1.79)	0.001	
**Female**				0.001
No. of participants	217,088	15,920		
No. of incident psoriasis	1,981	205		
**Hazard ratio for incident psoriasis (95% CI, *P*-value)**				
Unadjusted Model	reference	1.40 (1.21-1.61)	<0.001	
Adjusted Model 1	reference	1.40 (1.21-1.62)	<0.001	
Adjusted Model 2	reference	1.35 (1.17-1.56)	<0.001	
Adjusted Model 3	reference	1.30 (1.12-1.52)	<0.001	

Note: Adjusted Model 1: Age and sex were adjusted; Adjusted Model 2: Ethnicity, educational level, TDI, BMI, alcohol drinking, smoking status and IPAQ were additionally adjusted; Adjusted Model 3: T2DM, NSAID use and CRP were additionally adjusted.

Abbreviations: IBS, irritable bowel syndrome; FDR, false discovery rate; CI, confidence interval; TDI, Townsend Deprivation Index; BMI, Body Mass Index; IPAQ, International Physical Activity Questionnaire; T2DM, type 2 diabetes mellitus; NSAID, non-steroidal anti-inflammatory drug; CRP, C-reactive protein.

Table S4. Association between baseline IBS and risk of incident psoriasis by TDI.

Subgroup	IBS		*P*-value	P_{FDR}
	No	Yes		
**Low**				0.047
No. of participants	207,243	11,068		
No. of incident psoriasis	1,873	124		
**Hazard ratio for incident psoriasis (95% CI, *P*-value)**				
Unadjusted Model	reference	1.25 (1.04-1.50)	0.017	
Adjusted Model 1	reference	1.29 (1.07-1.55)	0.006	
Adjusted Model 2	reference	1.27 (1.06-1.53)	0.010	
Adjusted Model 3	reference	1.22 (1.01-1.48)	0.039	
**High**				<0.001
No. of participants	207,645	10,660		
No. of incident psoriasis	2,151	167		
**Hazard ratio for incident psoriasis (95% CI, *P*-value)**				
Unadjusted Model	reference	1.44 (1.23-1.68)	<0.001	
Adjusted Model 1	reference	1.52 (1.29-1.78)	<0.001	
Adjusted Model 2	reference	1.47 (1.25-1.72)	<0.001	
Adjusted Model 3	reference	1.46 (1.23-1.72)	<0.001	

Note: Median value of TDI (-2.12) was set as cutoff value, with TDI ≤ -2.12 considered as low group and TDI > -2.12 as high group.

Adjusted Model 1: Age and sex were adjusted; Adjusted Model 2: Ethnicity, educational level, TDI, BMI, alcohol drinking, smoking status and IPAQ were additionally adjusted; Adjusted Model 3: T2DM, NSAID use and CRP were additionally adjusted.

Abbreviations: IBS, irritable bowel syndrome; TDI, Townsend Deprivation Index; FDR, false discovery rate; CI, confidence interval; BMI, Body Mass Index; IPAQ, International Physical Activity Questionnaire; T2DM, type 2 diabetes mellitus; NSAID, non-steroidal anti-inflammatory drug; CRP, C-reactive protein.

Table S5. Association between baseline IBS and risk of incident psoriasis by BMI.

Subgroup	IBS		*P*-value	P_{FDR}
	No	Yes		
**< 25kg/m²**				0.185
No. of participants	135,142	7,775		
No. of incident psoriasis	1,040	74		
**Hazard ratio for incident psoriasis (95% CI, *P*-value)**				
Unadjusted Model	reference	1.23 (0.97-1.55)	0.090	
Adjusted Model 1	reference	1.26 (0.99-1.60)	0.057	
Adjusted Model 2	reference	1.24 (0.98-1.57)	0.079	
Adjusted Model 3	reference	1.19 (0.93-1.52)	0.177	
**≥ 25kg/m²**				<0.001
No. of participants	277,658	13,868		
No. of incident psoriasis	2,961	218		
**Hazard ratio for incident psoriasis (95% CI, *P*-value)**				
Unadjusted Model	reference	1.43 (1.25-1.64)	<0.001	
Adjusted Model 1	reference	1.48 (1.29-1.70)	<0.001	
Adjusted Model 2	reference	1.44 (1.26-1.66)	<0.001	
Adjusted Model 3	reference	1.41 (1.22-1.63)	<0.001	

Note: Adjusted Model 1: Age and sex were adjusted; Adjusted Model 2: Ethnicity, educational level, TDI, BMI, alcohol drinking, smoking status and IPAQ were additionally adjusted; Adjusted Model 3: T2DM, NSAID use and CRP were additionally adjusted.

Abbreviations: IBS, irritable bowel syndrome; BMI, Body Mass Index; FDR, false discovery rate; CI, confidence interval; TDI, Townsend Deprivation Index; IPAQ, International Physical Activity Questionnaire; T2DM, type 2 diabetes mellitus; NSAID, non-steroidal anti-inflammatory drug; CRP, C-reactive protein.

Table S6. Association between baseline IBS and risk of incident psoriasis by alcohol drinking.

Subgroup	IBS		*P*-value	P_{FDR}
	No	Yes		
**Never/Previous**				0.135
No. of participants	32,729	2,115		
No. of incident psoriasis	330	30		
**Hazard ratio for incident psoriasis (95% CI, *P*-value)**				
Unadjusted Model	reference	1.33 (0.91-1.93)	0.139	
Adjusted Model 1	reference	1.36 (0.94-1.99)	0.105	
Adjusted Model 2	reference	1.30 (0.89-1.90)	0.170	
Adjusted Model 3	reference	1.35 (0.92-1.99)	0.122	
**Current**				<0.001
No. of participants	381,265	19,569		
No. of incident psoriasis	3,684	262		
**Hazard ratio for incident psoriasis (95% CI, *P*-value)**				
Unadjusted Model	reference	1.36 (1.20-1.54)	<0.001	
Adjusted Model 1	reference	1.42 (1.25-1.61)	<0.001	
Adjusted Model 2	reference	1.39 (1.22-1.58)	<0.001	
Adjusted Model 3	reference	1.35 (1.18-1.54)	<0.001	

Note: Adjusted Model 1: Age and sex were adjusted; Adjusted Model 2: Ethnicity, educational level, TDI, BMI, alcohol drinking, smoking status and IPAQ were additionally adjusted; Adjusted Model 3: T2DM, NSAID use and CRP were additionally adjusted.

Abbreviations: IBS, irritable bowel syndrome; FDR, false discovery rate; CI, confidence interval; TDI, Townsend Deprivation Index; BMI, Body Mass Index; IPAQ, International Physical Activity Questionnaire; T2DM, type 2 diabetes mellitus; NSAID, non-steroidal anti-inflammatory drug; CRP, C-reactive protein.

Table S7. Association between baseline IBS and risk of incident psoriasis by smoking status.

Subgroup	IBS		*P*-value	P_{FDR}
	No	Yes		
**Never**				0.003
No. of participants	228,828	12,263		
No. of incident psoriasis	1,775	133		
**Hazard ratio for incident psoriasis (95% CI, *P*-value)**				
Unadjusted Model	reference	1.39 (1.16-1.66)	<0.001	
Adjusted Model 1	reference	1.41 (1.18-1.69)	<0.001	
Adjusted Model 2	reference	1.39 (1.16-1.66)	<0.001	
Adjusted Model 3	reference	1.35 (1.12-1.62)	0.002	
**Previous/Current**				0.001
No. of participants	184,121	9,370		
No. of incident psoriasis	2,229	158		
**Hazard ratio for incident psoriasis (95% CI, *P*-value)**				
Unadjusted Model	reference	1.34 (1.14-1.57)	<0.001	
Adjusted Model 1	reference	1.40 (1.19-1.65)	<0.001	
Adjusted Model 2	reference	1.37 (1.17-1.62)	<0.001	
Adjusted Model 3	reference	1.35 (1.14-1.60)	<0.001	

Note: Adjusted Model 1: Age and sex were adjusted; Adjusted Model 2: Ethnicity, educational level, TDI, BMI, alcohol drinking, smoking status and IPAQ were additionally adjusted; Adjusted Model 3: T2DM, NSAID use and CRP were additionally adjusted.

Abbreviations: IBS, irritable bowel syndrome; FDR, false discovery rate; CI, confidence interval; TDI, Townsend Deprivation Index; BMI, Body Mass Index; IPAQ, International Physical Activity Questionnaire; T2DM, type 2 diabetes mellitus; NSAID, non-steroidal anti-inflammatory drug; CRP, C-reactive protein.

Table S8. Association between baseline IBS and risk of incident psoriasis by IPAQ.

Subgroup	IBS		*P*-value	P_{FDR}
	No	Yes		
**Low/Moderate**				<0.001
No. of participants	188,378	9,889		
No. of incident psoriasis	1,795	146		
**Hazard ratio for incident psoriasis (95% CI, *P*-value)**				
Unadjusted Model	reference	1.49 (1.26-1.76)	<0.001	
Adjusted Model 1	reference	1.56 (1.32-1.85)	<0.001	
Adjusted Model 2	reference	1.54 (1.30-1.82)	<0.001	
Adjusted Model 3	reference	1.50 (1.25-1.79)	<0.001	
**High**				0.198
No. of participants	131,727	6,217		
No. of incident psoriasis	1,196	68		
**Hazard ratio for incident psoriasis (95% CI, *P*-value)**				
Unadjusted Model	reference	1.17 (0.92-1.49)	0.208	
Adjusted Model 1	reference	1.23 (0.96-1.58)	0.095	
Adjusted Model 2	reference	1.21 (0.95-1.55)	0.121	
Adjusted Model 3	reference	1.18 (0.92-1.53)	0.198	

Note: Adjusted Model 1: Age and sex were adjusted; Adjusted Model 2: Ethnicity, educational level, TDI, BMI, alcohol drinking, smoking status and IPAQ were additionally adjusted; Adjusted Model 3: T2DM, NSAID use and CRP were additionally adjusted.

Abbreviations: IBS, irritable bowel syndrome; IPAQ, International Physical Activity Questionnaire; FDR, false discovery rate; CI, confidence interval; TDI, Townsend Deprivation Index; BMI, Body Mass Index; T2DM, type 2 diabetes mellitus; NSAID, non-steroidal anti-inflammatory drug; CRP, C-reactive protein.

Table S9. Association between baseline IBS and risk of incident psoriasis by depression/anxiety.

Subgroup	IBS		*P*-value	P_{FDR}
	No	Yes		
**Without depression/anxiety**				0.001
No. of participants	375,128	16,519		
No. of incident psoriasis	3,458	204		
**Hazard ratio for incident psoriasis (95% CI, *P*-value value)**				
Unadjusted Model	reference	1.29 (1.12-1.48)	<0.001	
Adjusted Model 1	reference	1.35 (1.17-1.55)	<0.001	
Adjusted Model 2	reference	1.33 (1.15-1.53)	<0.001	
Adjusted Model 3	reference	1.31 (1.13-1.51)	<0.001	
**With depression/anxiety**				0.135
No. of participants	40,294	5,229		
No. of incident psoriasis	575	88		
**Hazard ratio for incident psoriasis (95% CI, *P*-value)**				
Unadjusted Model	reference	1.19 (0.95-1.49)	0.131	
Adjusted Model 1	reference	1.21 (0.97-1.52)	0.094	
Adjusted Model 2	reference	1.22 (0.97-1.53)	0.089	
Adjusted Model 3	reference	1.21 (0.95-1.53)	0.118	

Note: Adjusted Model 1: Age and sex were adjusted; Adjusted Model 2: Ethnicity, educational level, TDI, BMI, alcohol drinking, smoking status and IPAQ were additionally adjusted; Adjusted Model 3: T2DM, NSAID use and CRP were additionally adjusted.

Abbreviations: IBS, irritable bowel syndrome; FDR, false discovery rate; CI, confidence interval; TDI, Townsend Deprivation Index; BMI, Body Mass Index; IPAQ, International Physical Activity Questionnaire; T2DM, type 2 diabetes mellitus; NSAID, non-steroidal anti-inflammatory drug; CRP, C-reactive protein.

Table S10. Association between baseline IBS and risk of incident psoriasis by NSAID use.

Subgroup	IBS		*P*-value	P_{FDR}
	No	Yes		
**No**				<0.001
No. of participants	254,052	11,459		
No. of incident psoriasis	2,219	140		
**Hazard ratio for incident psoriasis (95% CI, *P*-value)**				
Unadjusted Model	reference	1.38 (1.17-1.64)	<0.001	
Adjusted Model 1	reference	1.43 (1.20-1.69)	<0.001	
Adjusted Model 2	reference	1.39 (1.17-1.66)	<0.001	
Adjusted Model 3	reference	1.40 (1.18-1.68)	<0.001	
**Yes**				0.006
No. of participants	161,368	10,289		
No. of incident psoriasis	1,814	152		
**Hazard ratio for incident psoriasis (95% CI, *P*-value)**				
Unadjusted Model	reference	1.26 (1.06-1.48)	0.007	
Adjusted Model 1	reference	1.33 (1.13-1.57)	<0.001	
Adjusted Model 2	reference	1.32 (1.12-1.56)	0.001	
Adjusted Model 3	reference	1.29 (1.09-1.54)	0.004	

Note: Adjusted Model 1: Age and sex were adjusted; Adjusted Model 2: Ethnicity, educational level, TDI, BMI, alcohol drinking, smoking status and IPAQ were additionally adjusted; Adjusted Model 3: T2DM, NSAID use and CRP were additionally adjusted.

Abbreviations: IBS, irritable bowel syndrome; NSAID, non-steroidal anti-inflammatory drug; FDR, false discovery rate; CI, confidence interval; TDI, Townsend Deprivation Index; BMI, Body Mass Index; IPAQ, International Physical Activity Questionnaire; T2DM, type 2 diabetes mellitus; CRP, C-reactive protein.

Table S11. Association between baseline IBS and risk of incident psoriasis by CRP.

Subgroup	IBS		*P*-value	P_{FDR}
	No	Yes		
**≤ 8mg/L**				<0.001
No. of participants	366,444	19,038		
No. of incident psoriasis	3,400	239		
**Hazard ratio for incident psoriasis (95% CI, *P*-value)**				
Unadjusted Model	reference	1.31 (1.15-1.49)	<0.001	
Adjusted Model 1	reference	1.37 (1.20-1.56)	<0.001	
Adjusted Model 2	reference	1.35 (1.18-1.54)	<0.001	
Adjusted Model 3	reference	1.32 (1.16-1.51)	<0.001	
**> 8mg/L**				0.047
No. of participants	21,200	1,322		
No. of incident psoriasis	313	31		
**Hazard ratio for incident psoriasis (95% CI, *P*-value)**				
Unadjusted Model	reference	1.47 (1.01-2.12)	0.043	
Adjusted Model 1	reference	1.57 (1.08-2.28)	0.017	
Adjusted Model 2	reference	1.51 (1.04-2.19)	0.030	
Adjusted Model 3	reference	1.48 (1.02-2.16)	0.038	

Note: Adjusted Model 1: Age and sex were adjusted; Adjusted Model 2: Ethnicity, educational level, TDI, BMI, alcohol drinking, smoking status and IPAQ were additionally adjusted; Adjusted Model 3: T2DM, NSAID use and CRP were additionally adjusted.

Abbreviations: IBS, irritable bowel syndrome; CRP, C-reactive protein; FDR, false discovery rate; CI, confidence interval; TDI, Townsend Deprivation Index; BMI, Body Mass Index; IPAQ, International Physical Activity Questionnaire; T2DM, type 2 diabetes mellitus; NSAID, non-steroidal anti-inflammatory drug.

Table S12. Association between baseline IBS and risk of incident psoriasis by PRS.

Subgroup	IBS		*P*-value	P_{FDR}
	No	Yes		
**Low risk**				0.003
No. of participants	201,081	10,589		
No. of incident psoriasis	1,432	113		
**Hazard ratio for incident psoriasis (95% CI, *P*-value)**				
Unadjusted Model	reference	1.46 (1.21-1.77)	<0.001	
Adjusted Model 1	reference	1.50 (1.24-1.82)	<0.001	
Adjusted Model 2	reference	1.49 (1.23-1.80)	<0.001	
Adjusted Model 3	reference	1.37 (1.12-1.67)	0.002	
**High risk**				0.033
No. of participants	201,141	10,522		
No. of incident psoriasis	2,438	171		
**Hazard ratio for incident psoriasis (95% CI, *P*-value)**				
Unadjusted Model	reference	1.27 (1.09-1.49)	0.002	
Adjusted Model 1	reference	1.35 (1.15-1.57)	<0.001	
Adjusted Model 2	reference	1.32 (1.13-1.54)	<0.001	
Adjusted Model 3	reference	1.21 (1.02-1.42)	0.024	

Note: Median value of psoriasis PRS (-0.35) was set as cutoff value, with PRS ≤ -0.35 considered as low risk group and PRS > -0.35 as high risk group.

Adjusted Model 1: Age and sex were adjusted; Adjusted Model 2: Ethnicity, educational level, TDI, BMI, alcohol drinking, smoking status and IPAQ were additionally adjusted; Adjusted Model 3: T2DM, NSAID use and CRP were additionally adjusted.

Abbreviations: IBS, irritable bowel syndrome; PRS, polygenic risk score; FDR, false discovery rate; CI, confidence interval; TDI, Townsend Deprivation Index; BMI, Body Mass Index; IPAQ, International Physical Activity Questionnaire; T2DM, type 2 diabetes mellitus; NSAID, non-steroidal anti-inflammatory drug; CRP, C-reactive protein.

Table S13. Risk of incident psoriasis associated with IBS duration

IBS duration	No	⩽10 years	>10 years	*P*-value
No. of participants	415,422	10,649	11,099	
No. of incident psoriasis	4,033	160	132	
Follow-up, person-years	5,817,643	150,290	157,106	
Follow-up, years Median, IQR	14.4 (13.6, 15.1)	14.4 (13.7, 15.2)	14.4 (13.7, 15.2)	
Hazard ratio for incident psoriasis (95% CI, *P*-value)				
Unadjusted Model	reference	1.51 (1.29-1.77)	1.20 (1.01-1.43)	<0.001
Adjusted Model 1	reference	1.59 (1.36-1.86)	1.24 (1.04-1.48)	<0.001
Adjusted Model 2	reference	1.54 (1.32-1.81)	1.23 (1.04-1.47)	<0.001
Adjusted Model 3	reference	1.45 (1.22-1.71)	1.26 (1.06-1.51)	<0.001

Note: Adjusted Model 1: Age and sex were adjusted; Adjusted Model 2: Ethnicity, education level, TDI, BMI, alcohol drinking, smoking status and IPAQ were additionally adjusted; Adjusted Model 3: T2DM, NSAID use and CRP were additionally adjusted.

Abbreviations: IBS, irritable bowel syndrome; IQR: inter-quartile range; CI, confidence interval; TDI, Townsend Deprivation Index; BMI, Body Mass Index; IPAQ, International Physical Activity Questionnaire; T2DM, type 2 diabetes mellitus; NSAID, non-steroidal anti-inflammatory drug; CRP, C-reactive protein.

STROBE Statement—checklist of items that should be included in reports of observational studies

	Item No.	Recommendation	Page No.
**Title and abstract**	1	(*a*) Indicate the study's design with a commonly used term in the title or the abstract	1
		(*b*) Provide in the abstract an informative and balanced summary of what was done and what was found	1
**Introduction**			
Background/rationale	2	Explain the scientific background and rationale for the investigation being reported	2
Objectives	3	State specific objectives, including any prespecified hypotheses	3
**Methods**			
Study design	4	Present key elements of study design early in the paper	3
Setting	5	Describe the setting, locations, and relevant dates, including periods of recruitment, exposure, follow-up, and data collection	3
Participants	6	(*a*) *Cohort study*—Give the eligibility criteria, and the sources and methods of selection of participants. Describe methods of follow-up<br>*Case-control study*—Give the eligibility criteria, and the sources and methods of case ascertainment and control selection. Give the rationale for the choice of cases and controls<br>*Cross-sectional study*—Give the eligibility criteria, and the sources and methods of selection of participants	3
		(*b*) *Cohort study*—For matched studies, give matching criteria and number of exposed and unexposed<br>*Case-control study*—For matched studies, give matching criteria and the number of controls per case	
Variables	7	Clearly define all outcomes, exposures, predictors, potential confounders, and effect modifiers. Give diagnostic criteria, if applicable	3-4
Data sources/ measurement	8*	For each variable of interest, give sources of data and details of methods of assessment (measurement). Describe comparability of assessment methods if there is more than one group	3-4
Bias	9	Describe any efforts to address potential sources of bias	5
Study size	10	Explain how the study size was arrived at	3

Continued on next page

Quantitative variables	11	Explain how quantitative variables were handled in the analyses. If applicable, describe which groupings were chosen and why	3-4
Statistical methods	12	(*a*) Describe all statistical methods, including those used to control for confounding	4-5
		(*b*) Describe any methods used to examine subgroups and interactions	5
		(*c*) Explain how missing data were addressed	5
		(*d*) *Cohort study*—If applicable, explain how loss to follow-up was addressed<br>*Case-control study*—If applicable, explain how matching of cases and controls was addressed<br>*Cross-sectional study*—If applicable, describe analytical methods taking account of sampling strategy	4
		(*e*) Describe any sensitivity analyses	5
**Results**			
Participants	13*	(a) Report numbers of individuals at each stage of study—eg numbers potentially eligible, examined for eligibility, confirmed eligible, included in the study, completing follow-up, and analysed	6
		(b) Give reasons for non-participation at each stage	Fig. 1
		(c) Consider use of a flow diagram	Fig. 1
Descriptive data	14*	(a) Give characteristics of study participants (eg demographic, clinical, social) and information on exposures and potential confounders	6, Tab. 1
		(b) Indicate number of participants with missing data for each variable of interest	Tab. 1
		(c) *Cohort study*—Summarise follow-up time (eg, average and total amount)	6
Outcome data	15*	*Cohort study*—Report numbers of outcome events or summary measures over time	6
		Case-control study—Report numbers in each exposure category, or summary measures of exposure	N/A
		Cross-sectional study—Report numbers of outcome events or summary measures	N/A
Main results	16	(*a*) Give unadjusted estimates and, if applicable, confounder-adjusted estimates and their precision (eg, 95% confidence interval). Make clear which confounders were adjusted for and why they were included	6
		(*b*) Report category boundaries when continuous variables were categorized	6
		(*c*) If relevant, consider translating estimates of relative risk into absolute risk for a meaningful time period	N/A

Continued on next page

Other analyses	17	Report other analyses done—eg analyses of subgroups and interactions, and sensitivity analyses	6-7
**Discussion**			
Key results	18	Summarise key results with reference to study objectives	7
Limitations	19	Discuss limitations of the study, taking into account sources of potential bias or imprecision. Discuss both direction and magnitude of any potential bias	9-10
Interpretation	20	Give a cautious overall interpretation of results considering objectives, limitations, multiplicity of analyses, results from similar studies, and other relevant evidence	7-10
Generalisability	21	Discuss the generalisability (external validity) of the study results	7
**Other information**			
Funding	22	Give the source of funding and the role of the funders for the present study and, if applicable, for the original study on which the present article is based	10

*Give information separately for cases and controls in case-control studies and, if applicable, for exposed and unexposed groups in cohort and cross-sectional studies.

Table S12. Risk of incident psoriasis associated with IBS duration

IBS duration	No	⩽10 years	>10 years	*P*-value
No. of participants	415,422	10,649	11,099	
No. of incident psoriasis	4,033	160	132	
Follow-up, person-years	5,817,643	150,290	157,106	
Follow-up, years Median, IQR	14.4 (13.6, 15.1)	14.4 (13.7, 15.2)	14.4 (13.7, 15.2)	
Hazard ratio for incident psoriasis (95% CI, *P*-value)				
Unadjusted Model	reference	1.51 (1.29-1.77)	1.20 (1.01-1.43)	<0.001
Adjusted Model 1	reference	1.59 (1.36-1.86)	1.24 (1.04-1.48)	<0.001
Adjusted Model 2	reference	1.54 (1.32-1.81)	1.23 (1.04-1.47)	<0.001
Adjusted Model 3	reference	1.45 (1.22-1.71)	1.26 (1.06-1.51)	<0.001

Note: Adjusted Model 1: Age and sex were adjusted; Adjusted Model 2: Ethnicity, education level, TDI, BMI, alcohol drinking, smoking status and IPAQ were additionally adjusted; Adjusted Model 3: T2DM, NSAID use and CRP were additionally adjusted.

Abbreviations: IBS, irritable bowel syndrome; IQR: inter-quartile range; CI, confidence interval; TDI, Townsend Deprivation Index; BMI, Body Mass Index; IPAQ, International Physical Activity Questionnaire; T2DM, type 2 diabetes mellitus; NSAID, non-steroidal anti-inflammatory drug; CRP, C-reactive protein.
